# Choledochoscope-guided treatment of pulmonary embolism caused by ventricular myxoma

**DOI:** 10.1186/s13019-023-02250-0

**Published:** 2023-04-17

**Authors:** Yong Mao, Cuiting Wang, Yalin Wei, Yongnan Li, Xiangyang Wu

**Affiliations:** 1 Department of Cardiac Surgery, Lanzhou University Second Hospital, Lanzhou, Gansu China; 2 Health Science Center of Lanzhou University, Lanzhou, Gansu China

**Keywords:** Right heart tumor, Pulmonary embolism, Tricuspid regurgitation, Choledochoscope, Case report

## Abstract

A 33-year male patient presented with a 6-month history of cough and shortness of breath upon physical activity. Echocardiography demonstrated right ventricular space-occupying lesions. Contrast-enhanced computed tomography of the chest showed multiple emboli in the pulmonary artery and its branches. Right ventricle tumor (myxoma) resection, tricuspid valve replacement, and clearance of the pulmonary artery thrombus were performed under cardiopulmonary bypass. Minimally invasive forceps and balloon urinary catheters were used to clear the thrombus. Clearance was confirmed by direct visualization using a choledochoscope. The patient recovered well and was discharged. The patient was prescribed oral warfarin 3 mg/day, and the international normalized ratio for prothrombin time was maintained between 2.0 and 3.0. Pre-discharge echocardiogram showed no lesion in the right ventricle or pulmonary arteries. The 6-month follow-up echocardiography indicated that the tricuspid valve was functioning well and showed no thrombus in the pulmonary artery.

## Introduction

Primary tumors of the heart are uncommon and mostly benign, with an overall incidence of 0.17–0.19% [1]. A right ventricular tumor can cause outflow obstruction and tricuspid valve regurgitation producing symptoms including palpitation, chest tightness, and edema. As most benign heart tumors are soft and loose, they can dislodge from the heart tissue. When a right ventricular tumor (e.g. myxoma) dislodges, it can cause a pulmonary embolism and pulmonary hypertension leading to dyspnea, chest pain, and hemoptysis. Resection of a right ventricular tumor together with thromboembolectomy and the use of a flexible fiber scope to guide and visualize clearance of a pulmonary embolism has been reported. In this case report, we describe the successful resection of a right ventricle myxoma, tricuspid valve replacement, and clearance of a pulmonary embolism using a balloon urinary catheter guided by a choledochoscope.

## Case report

### Patients and methods

**Fig. 1:**
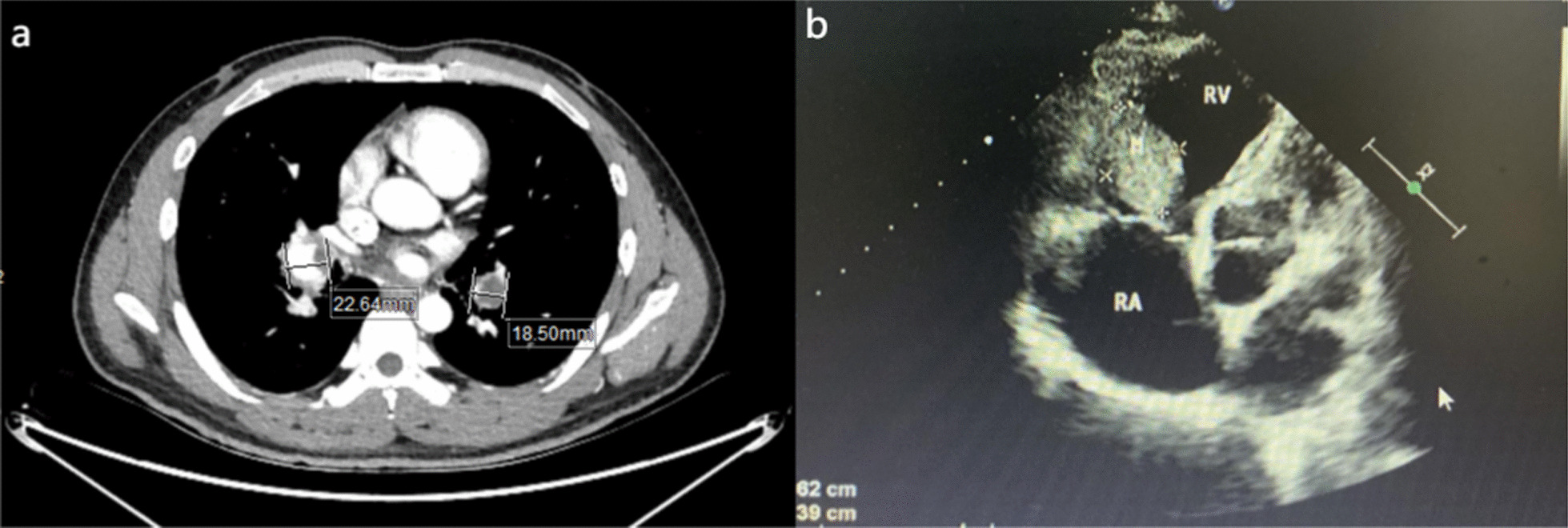
**a** Contrast-enhanced computed tomography showed emboli formation in the pulmonary artery and its branches; **b** Transthoracic echocardiography before surgery showed a right ventricular space-occupying lesion

A 33-year male presented with history of cough and shortness of breath for 6 months. The patients presented with the following: blood pressure = 133/89 mmHg, heart rate = 124 beats/min, respiratory rate = 20/min, and body temperature = 36.3 °C. Chest exam was normal. At admission the patient had a D-dimmer of 0.99 µg/Ml (range 0–0.5 µg/mL) and N-terminal pro B type natriuretic peptide level of 219 pg/ml (range 0–125 pg/ml). Contrast-enhanced computed tomography (CECT) of the chest showed an extensive emboli formation in the pulmonary artery and its branches, including left and right pulmonary arteries, and a soft tissue mass protruding into the right ventricle (Fig. 1). Echocardiography showed pulmonary hypertension (systolic pressure = 78 mmHg), right ventricular space-occupying lesion (31 × 34 mm), and moderate grade tricuspid regurgitation [2] (Fig. 1).

## Results

The patient underwent a median sternotomy under cardiopulmonary bypass. The ventricular tumor was resected, the tricuspid valve was replaced, and embolism was cleared successfully during the same procedure.

A longitudinal right atriotomy revealed a wide-base mass of 3 × 3 cm originating from the right ventricle anterior wall that was adhered to the regulatory fascicles, papillary muscles, chordae tendineae, and trabeculae. The right ventricle neoplasm was excised from its base and its affected tissue including the chordae tendineae and trabeculae. The anterior and posterior leaflets of the tricuspid valve were removed and replaced with a bioprosthetic valve (mosaic 29# biological valve).

**Fig. 2:**
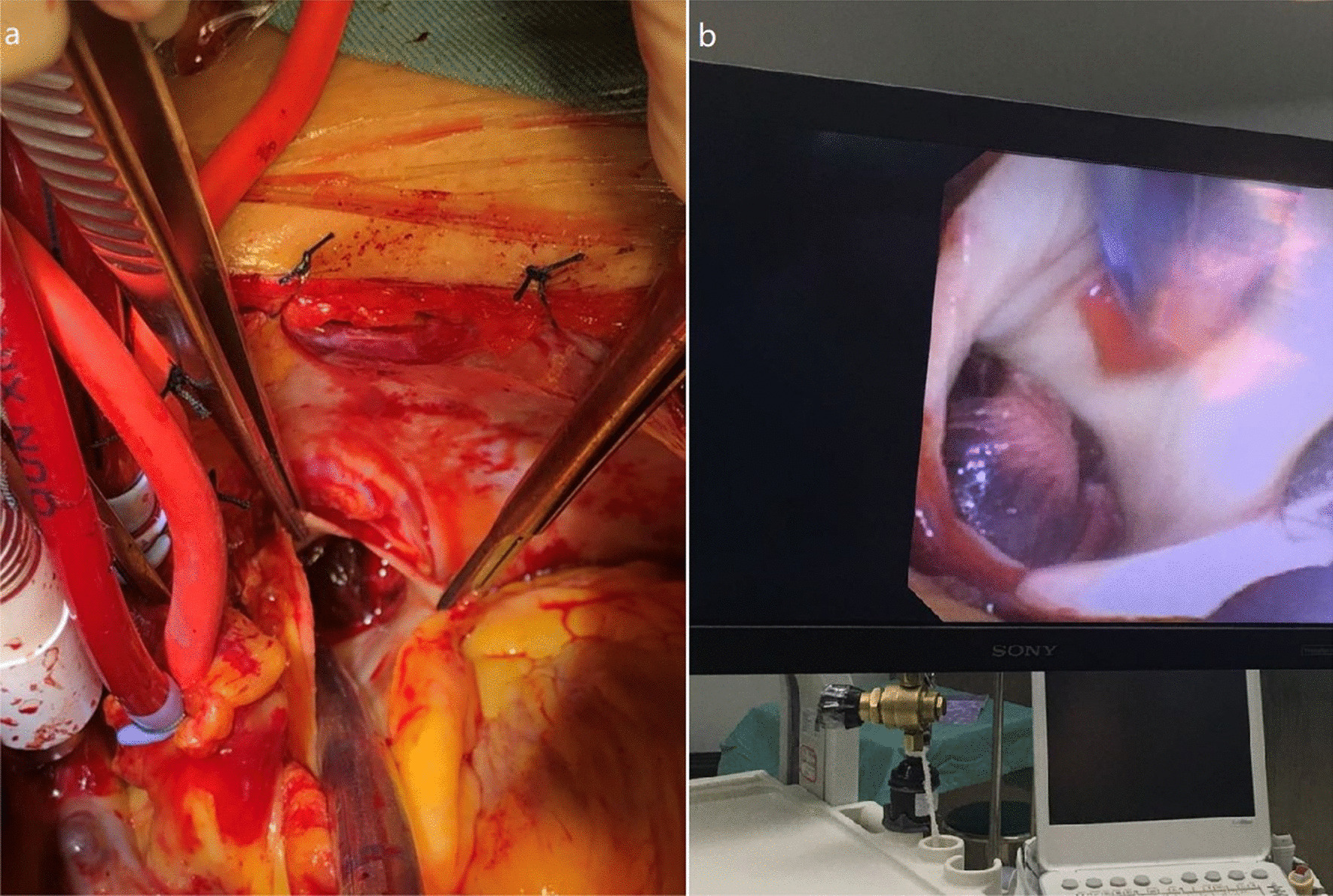
**a** Thrombus found in the pulmonary trunk; **b** Clearance of thrombus guided by Choledochoscope

**Fig. 3:**
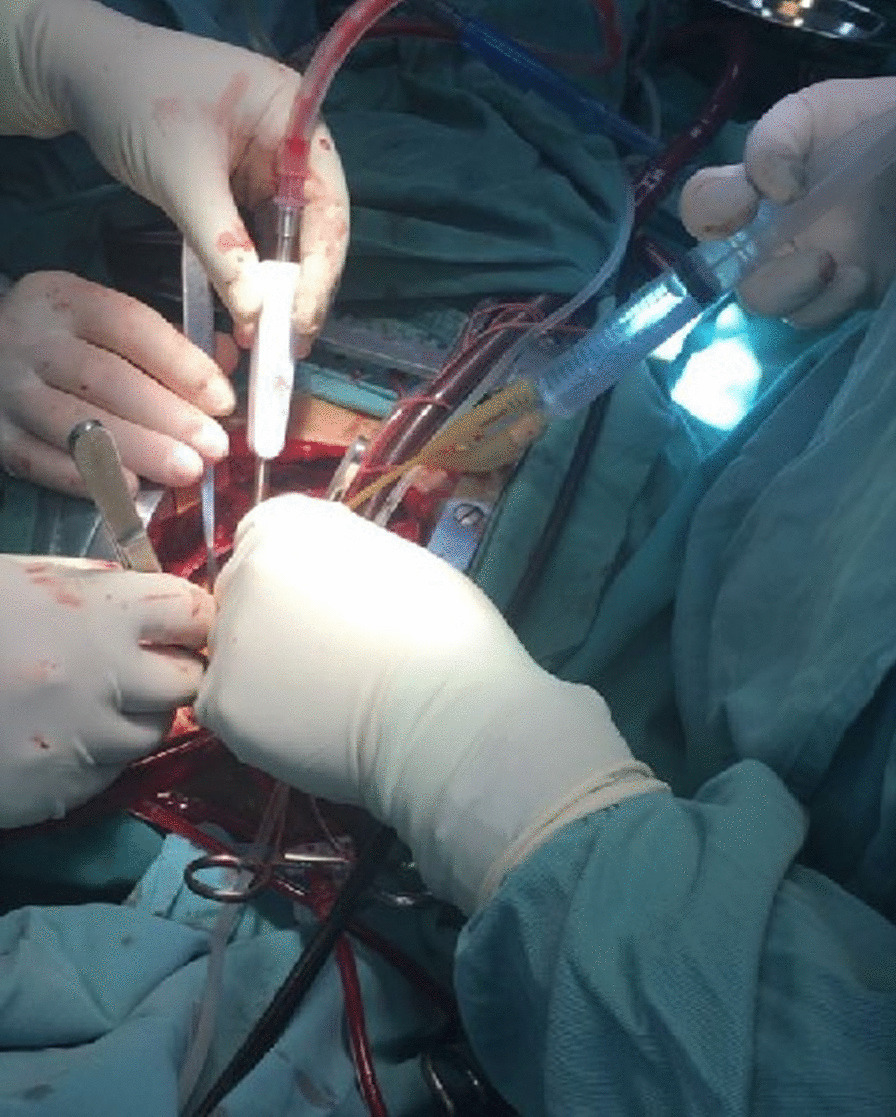
A #5 urinary catheter was placed in the distal end of the pulmonary artery

The thrombus in the pulmonary trunk and left and right branches was cleared using balloon urinary catheters and minimally invasive forceps (GEISTER, Germany). Clearance was confirmed under direct visualization using a choledochoscope (Olympus, America) (Fig. 2a). A 5F balloon urinary catheter (Folley) was used to clear the thrombus. The catheter was passed through the distal branch, the balloon was inflated, and the thrombus was pulled out (Fig. 3). The procedure was repeated until the thrombus was cleared.

**Fig. 4:**
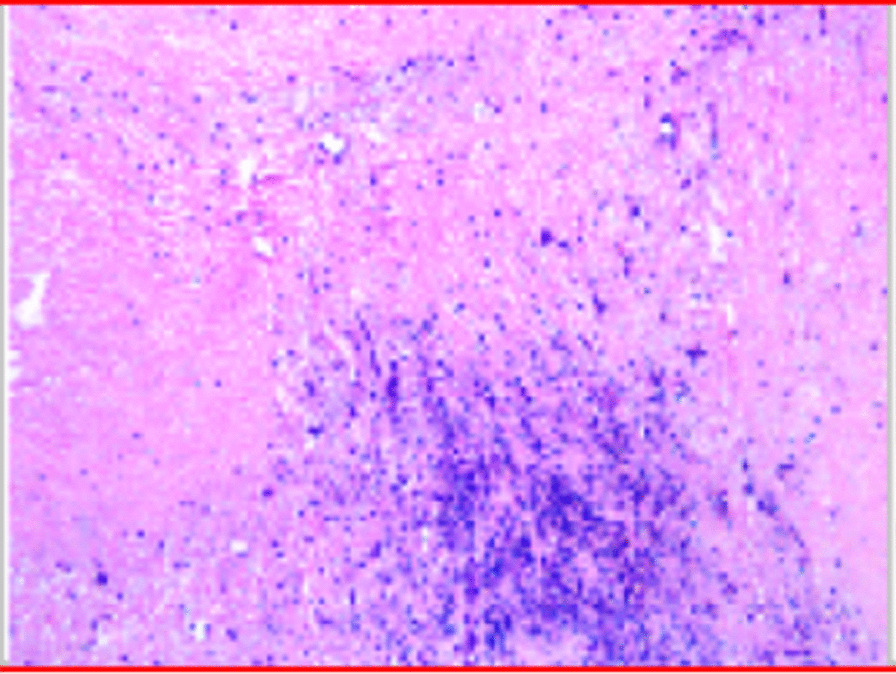
Tumor pedicle; pathology shows a myxoma

Histopathological diagnosis of the right ventricle mass was myxoma (Fig. 4). The patient recovered without any major event and was discharged with oral warfarin 3 mg/day. The international normalized ratio for prothrombin time was maintained between 2.0 and 3.0. The pre-discharge echocardiogram showed no mass in the right ventricular and no obstructive lesion on the pulmonary trunk or left and right pulmonary arteries. At the 6-month follow-up there was no obstructive lesion in the pulmonary arteries, and the patient’s general condition was good.

## Discussion

This case of right ventricular myxoma with tricuspid valve regurgitation and pulmonary artery embolism was successfully treated in a single setting with mass resection, valve replacement, and emboli clearance using a balloon catheter confirmed visually with a choledochoscope.

The incidence of heart tumors is 0.02%, and 75% of these tumors are benign. Myxoma is the most common type of benign tumor in adult patients with cardiac tumors, three-quarters of which are located in the left atrium. The incidence of right ventricular myxoma reported in the literature is 3–4%. The location and size of a myxoma determines its clinical manifestations with regards to embolism and obstruction [3, 4].

The patient presented in this report had a right ventricular myxoma on the anterior wall of the right ventricle with a pulmonary thrombus embolism. The traditional treatment of acute pulmonary embolism is anticoagulant thrombolysis and percutaneous catheter-directed therapy. However, there is no clear guideline for treating pulmonary embolisms combined with right ventricular tumors. One study reported the use of warfarin to treat a pulmonary embolism following incomplete surgical resection of a right ventricular myxoma, with no recurrence of emboli after 20 months [5]. Another case study reported resection of a right ventricular myxoma of 9.5 × 5.0 cm. However, resection of a ventricular tumor combined with clearance of a pulmonary embolism has not often been reported [6].

Pulmonary embolectomy has rarely been used to treat acute right ventricle dysfunction caused by a pulmonary embolism. However, the procedure and outcome of surgical pulmonary embolectomy have been greatly improved by the use of intraoperative flexible fiber scopes (i.e. fiberoptic bronchoscope, endoscope) to clear and directly visualize the pulmonary arteries [7–9]. In the present case report, we successfully used a choledochoscope to visualize and guide the clearance of a pulmonary tree thromboembolism.

In conclusion, a right ventricular myxoma involving the tricuspid valve and regurgitation leading to complications such as pulmonary artery embolism can be treated in a single setting. Tumor resection and removal of a thrombus using a balloon catheter and visual clearance of thrombus with a choledochoscope could be considered for patients with cardiac tumors.

## Data Availability

None.
